# TBK1 Kinase Addiction in Lung Cancer Cells Is Mediated via Autophagy of Tax1bp1/Ndp52 and Non-Canonical NF-κB Signalling

**DOI:** 10.1371/journal.pone.0050672

**Published:** 2012-11-29

**Authors:** Alice C. Newman, Caroline L. Scholefield, Alain J. Kemp, Michelle Newman, Edward G. McIver, Ahmad Kamal, Simon Wilkinson

**Affiliations:** 1 Edinburgh Cancer Research UK Centre, MRC Institute of Genetics and Molecular Medicine, University of Edinburgh, Edinburgh, United Kingdom; 2 Centre for Therapeutics Discovery, MRC Technology, London, United Kingdom; Johns Hopkins School of Medicine, United States of America

## Abstract

K-Ras dependent non-small cell lung cancer (NSCLC) cells are ‘addicted’ to basal autophagy that reprograms cellular metabolism in a lysosomal-sensitive manner. Here we demonstrate that the xenophagy-associated kinase TBK1 drives basal autophagy, consistent with its known requirement in K-Ras-dependent NSCLC proliferation. Furthermore, basal autophagy in this context is characterised by sequestration of the xenophagy cargo receptor Ndp52 and its paralogue Tax1bp1, which we demonstrate here to be a *bona fide* cargo receptor. Autophagy of these cargo receptors promotes non-canonical NF-κB signalling. We propose that this TBK1-dependent mechanism for NF-κB signalling contributes to autophagy addiction in K-Ras driven NSCLC.

## Introduction

A strategy to target tumour progression is to identify specific molecular vulnerabilities conferred by genetic background. Activation of K-Ras by mutation, or other means, renders non-small cell lung cancer (NSCLC) cells ‘addicted’ to the presence of TBK1 (TANK-binding kinase 1) protein for continued proliferation and/or survival [1], possibly via direct stimulation of TBK1 activity [2]. TBK1 and its paralogue, IKKε, alongside the well-characterised IKKα and IKKβ proteins, constitute a subfamily of serine-threonine protein kinases [3]. TBK1 and IKKε were originally described as mediating NF-κB transcription factor activation [4], [5], [6]. It has also been proposed that constitutive promotion of NF-κB signalling in NSCLC cells, downstream of K-Ras, could underlie TBK1 ‘addiction’ [1]. Indeed, gene expression profiling has shown that a K-Ras driven, NF-κB-like signature is dependent upon the TBK1 gene [1]. However, mechanistic evidence for TBK1-mediated NF-κB engagement in cancer has been sparse. Alternative explanations of TBK1 addiction have been proposed, such as direct activation of pro-survival Akt kinase signalling [7], [8]. However, no individual contribution of TBK1 is necessarily mutually exclusive with others.

A second pathway engaged downstream of K-Ras activation is macroautophagy (hereafter autophagy) [9], [10], [11]. Autophagy is a multistep lysosomal degradation process [12]. In the early stages, dependent upon the action of core autophagy genes such as *ATG5* and *ULK1*, double membraned vesicles decorated with ubiquitin-like proteins of the LC3/GABARAP family, called autophagosomes, begin to form. These nascent autophagosomes enclose around, and sequester, cytosol as they mature. Depending upon context, this can be a non-selective or selective process, the latter involving sequestration of discrete populations, or ‘cargoes’, of organelles, proteins or other structures, such as bacteria in the case of ‘xenophagy’. Selectivity is mediated via cargo receptors that link LC3/GABARAP proteins to the cargo, in presumed multi-molecular complexes [12]. Autophagy impacts on cell fate by both this selective sequestration of cargo complexes from the cytosol and via subsequent lysosomal degradation of the contents of autophagosome, which releases metabolites into the cytosol. This latter step is inhibited by lysosomotrophic drugs such as chloroquine. Such compounds are a potential class of agents for therapeutic manipulation of autophagy [11], [13].

The role of autophagy in acute response to Ras mutation is unclear. It has been demonstrated to act as an effector of survival, cell death or senescence [9], [14], [15]. However, continuous basal autophagy is unambiguously required for the proliferation and/or survival of established K-Ras driven cancer cells [10], [11], [13]. A role of autophagy here is direct regulation of cellular metabolism, via mitochondrial homoeostasis, protein quality control and/or remodelling of the cellular metabolite pool. Cumulatively, these mechanisms regulate the balance between oxidative phosphorylation and aerobic glycolysis [10], [11], [13]. Some of these mechanisms are dependent upon lysosomal degradation, as shown by sensitivity of metabolic effects to lysosomotrophic agents such as chloroquine. However, it has not been addressed how autophagy is engaged downstream of K-Ras. Additionally, it is not clear whether autophagy ‘addiction’ can be explained solely by direct metabolic effects or if sequestration of specific proteins modulates cell fate, via alteration of signal transduction within the cell.

Interestingly, TBK1 has been implicated in the autophagy of cytosolic *Salmonella spp.* bacteria from within cells [16]. The novel TBK1-binding protein and xenophagy cargo receptor, Ndp52, recognises ubiquitinated proteins, on the surface of bacteria, and carbohydrate-binding proteins on ruptured host vesicles [17], [18]. A non-redundant step in this pathway has recently been demonstrated to be the TBK1-mediated phosphorylation of a further novel cargo receptor, Optineurin [16]. The relevance of TBK1 signalling outside of xenophagy is unclear. However, we show here that basal autophagy, with some parallels to xenophagy and resulting in turnover of cargo receptors such as Ndp52 and the paralogous protein Tax1bp1, is constitutively engaged in TBK1-addicted NSCLC cells by the kinase activity of TBK1. We demonstrate a central role for this autophagy in driving non-canonical NF-κB signalling mediated via the RelB transcription factor. We propose that this pathway complements direct metabolic mechanisms in the contribution of basal autophagy to supporting proliferation and/or survival in NSCLC cells. Our findings thus expand the role of autophagy addiction in K-Ras driven cancer and show mechanistic interplay with the TBK1-NF-κB pathway.

## Results

### Autophagy in K-Ras Dependent Lung Cancer Cells is Downstream of TBK1 Kinase

**Figure 1:**
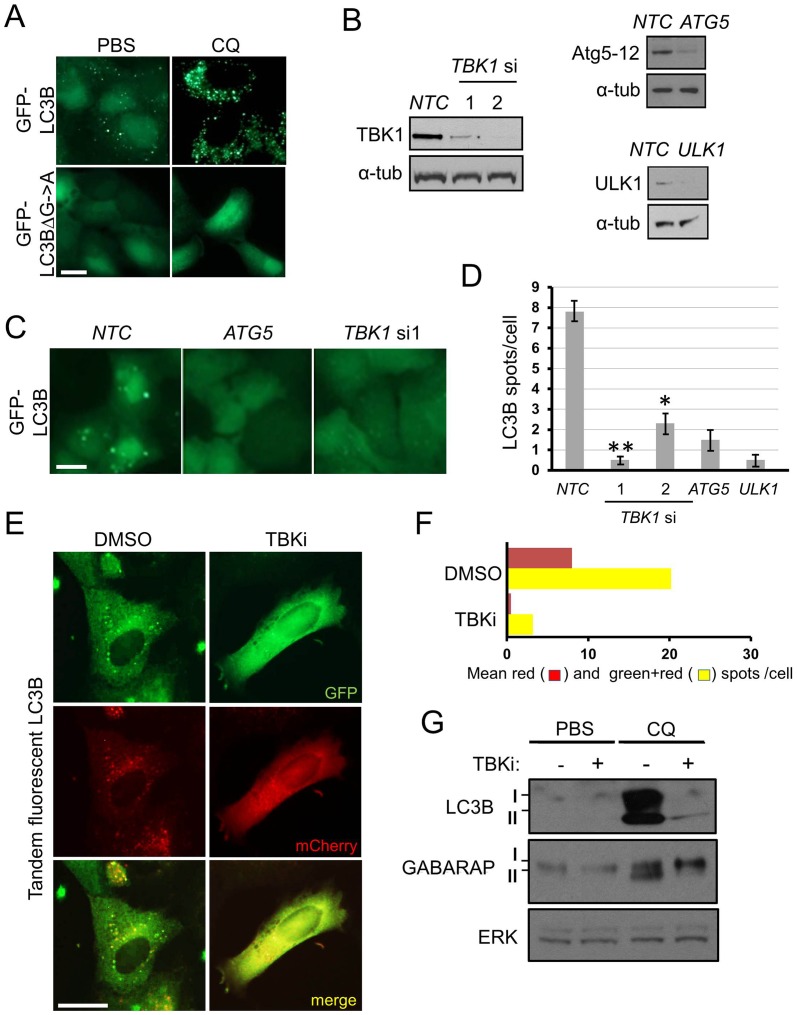
TBK1 kinase activity engages basal autophagy in A549 NSCLC cells. a) A549 GFP-LC3B or GFP-LC3BΔG->A cells were treated with PBS or 5 µM chloroquine (CQ) for 8 h and cells imaged for GFP. **b, c)** A549 GFP-LC3B cells were transfected with indicated siRNA for 72 h (*NTC* = non-targeting control) and b) cell extracts blotted for indicated proteins (α-tub = α-tubulin) or cells were c) imaged for GFP-LC3B puncta. **d)** A549 FLAG-HA-LC3B cells were transfected with indicated siRNA and HA-positive puncta immunostained, imaged and counted at 72 h (n = 3, ± S.E.M., * = p<0.05, ** = p<0.01). **e,f)** A549 tandem-fluorescent-LC3B (tfLC3B) cells were treated with DMSO or 1 µM MRT68601 (TBKi) for 24 h and e) imaged and f) quantified for total number of autophagic puncta (green+red) and the subset of these puncta with undetectable GFP signal (red), as described in detail in Materials and Methods. **g)** A549 cells were treated with DMSO or 10 µM MRT68601 (TBKi) for 24 h in the presence or absence of PBS or 5 µM (CQ) chloroquine and cell extracts blotted for indicated proteins. Scale bars = 50 µm in all panels.

To investigate the role of TBK1 in K-Ras driven basal autophagy we developed a NSCLC culture model in K-Ras ‘addicted’ A549 cells [1]. We found that the cytosol of these contained abundant punctate structures that labelled with GFP-LC3B fusion protein, but not with lipid-unconjugatable GFP-LC3BΔG->A (Fig. 1a). The abundance of GFP-LC3B puncta was dramatically elevated by chloroquine treatment (Fig. 1a). According to the methodology of the field [19], we then took these LC3B puncta to represent a basal, steady-state level of autophagosomes. These autophagosomes were absent when RNAi was used to knockdown the autophagy genes *ATG5* and *ULK1* (Fig. 1b-d). RNAi of *TBK1* produced similar effects, positioning this kinase upstream of basal autophagosome abundance (Fig. 1b-d). We extended these findings using autophagy flux assays in combination with a member of a recently described family of enzymatic inhibitors of TBK1 (MRT68601, hereafter referred to as TBKi, Fig. S1) [20]. Inhibitor treatment of A549 tandem-fluorescent LC3B cells [21] resulted in reduction of both early autophagic (‘green+red‘) LC3B puncta and acidic late autophagosomes (‘red’ puncta) (Fig. 1e, f). Correspondingly, chloroquine-mediated accumulation of lipidated LC3B-II or GABARAP-II, the form of these proteins correlated with formation of autophagosomes, was prevented by inhibitor treatment (Fig. 1g). Taken together, these data show that TBK1 kinase indeed promotes autophagy, acting at the early step of autophagosome formation, rather than repressing autophagosome maturation into the lysosomal acidic compartment [19].

### Basal Autophagy Targets the Cargo Receptors Ndp52 and Tax1bp1

**Figure 2:**
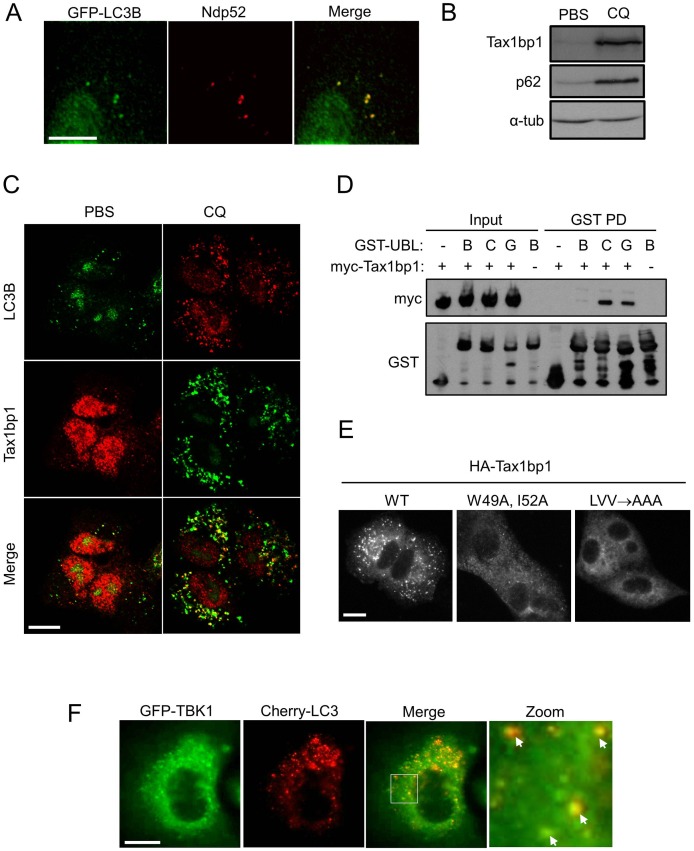
Basal autophagy targets TBK1-binding proteins Ndp52 and Tax1bp1. a) A549 GFP-LC3B cells were stained for Ndp52 and imaged. **b,c)** A549 cells were treated with PBS or 5 µM chloroquine (CQ) overnight and b) cell extracts blotted for indicated proteins (α-tub = alpha tubulin) or c) cells co-stained for Tax1bp1 and LC3B, and imaged by confocal microscopy. **d)** 293FT cells were transfected with myc-TAX1BP1 expression vector, or empty vector control, and lysates subjected to pull-down with indicated fusion protein, as described in Materials and Methods (- = GST only, B = GST-LC3B, C = GST-LC3C, G = GST-GABARAP). **e)** A549 FLAG-HA-TAX1BP1 cell lines stably expressing the indicated variants of Tax1bp1 protein (see text) were treated with 5****µM chloroquine overnight and stained for HA puncta. **f)** A549 GFP-TBK1 mCherry-LC3C cells were imaged (arrows indicate colocalisation). Scale bars = 25 µm in all panels.

Bacterial autophagy (xenophagy) is mediated via TBK1 and its binding partners, the xenophagy cargo receptors Ndp52 and Optineurin [16], [18]. In a parallel to this, we found recruitment of Ndp52, and the Ndp52 paralogue Tax1bp1, to basal autophagosomes in A549 cells (Fig. 2a, c). We also demonstrated a constitutive Ndp52-TBK1 complex and confirmed a previous report that Tax1bp1 could bind TBK1 [22] (Fig. S2a, b). These data imply that Ndp52 and Tax1bp1 may be being targeted for sequestration by basal autophagy. Accordingly, chloroquine treatment stabilised Tax1bp1 similar to a positive control, the p62 cargo receptor (Fig. 2b), and concomitant with increased Tax1bp1 localisation to LC3B-positive vesicles (Fig. 2c). Ubiquitin-like proteins of the LC3 and GABARAP subfamilies are capable of binding Tax1bp1, with varying affinities (Fig. 2d). This suggests that Tax1bp1 is, like, Ndp52, a *bona fide* cargo receptor protein. Further support for this finding comes from the observation that deletion of putative LC3/GABARAP family binding motifs in Tax1bp1 ablates localisation to autophagosomes (Fig. 2e). These motifs include both an ‘canonical’ LIR (LC3-interacting region) motif (W49, V50, G51, I52) and a ‘non-canonical LIR’ motif, the latter inferred from that recently described for Ndp52 binding to LC3C (L141, V142, V143) [23] (Fig. 2e, also see alignments in Fig. S2c). Consistent with the role of TBK1 in the above processes, TBK1 protein was also shown to localise to basal autophagosomes (Fig. 2f).

### TBK1 Drives Non-canonical NF-κB Signalling by Autophagic Sequestration of Ndp52 and Tax1bp1

**Figure 3:**
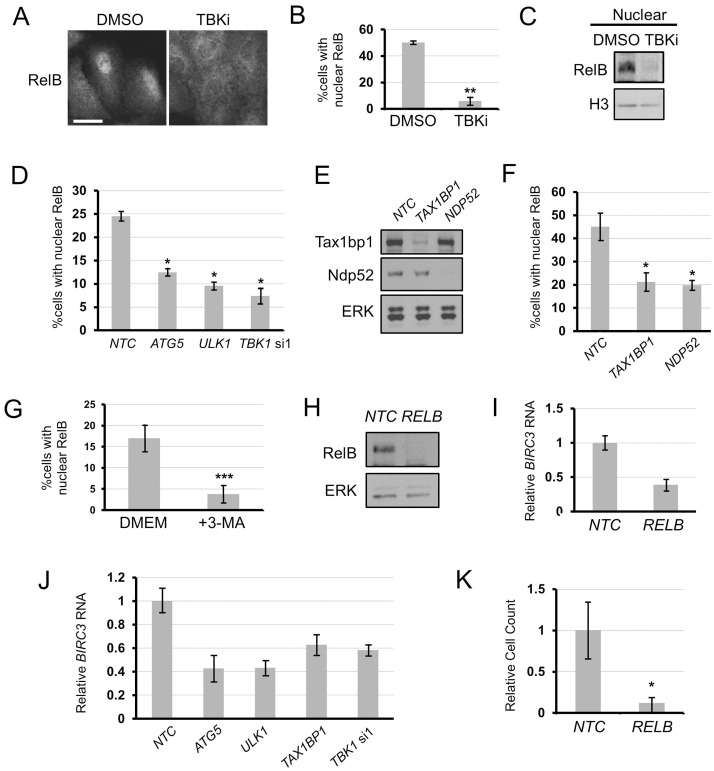
TBK1, autophagy and the Tax1bp1/Ndp52 cargo receptors maintain pro-survival/proliferation non-canonical NF-κB signalling in A549 NSCLC cells. a–c) A549 cells were treated overnight with DMSO or 10 µM MRT68601 (TBKi) and a) stained for RelB (scale bar = 50 µm), nuclear localisation quantified in b) (n = 3, ± S.E.M., ** = p<0.01) or c) nuclear extracts prepared and blotted for indicated proteins. **d–f)** A549 cells were transfected with indicated siRNA (*NTC* = non-targeting control) for 72 h and e) cell extracts blotted for indicated proteins or d,f) cells were stained and quantified for nuclear RelB (n = 3, ± S.E.M., * = p<0.05). **g)** A549 cells were treated with PBS or 10 mM 3-methyladenine (3-MA) for 24 hours and then stained and quantified for nuclear RelB (n = 3, ± S.E.M., *** = p<0.005). **h,i)** A549 cells were infected with indicated lentivirus and, at 72 h post infection, h) cell extracts blotted for indicated proteins or i) total RNA quantified for *BIRC3* mRNA (n = 3; ± S.D.). **j)** A549 cells were transfected with indicated siRNA for 72 h and total RNA extracts were subjected to qRT-PCR for *BIRC3* mRNA (n = 3; ± S.D.). **k)** A549 cells were infected with indicated lentivirus and at 120 h post infection cell number counted as described in Materials and Methods (n = 3, ± S.E.M., * = p<0.05).

We next hypothesised a link between TBK1-driven autophagy and basal NF-κB signalling. Nuclear localisation of canonical NF-κB pathway subunits c-Rel or RelA was not detectable in the A549 cell line (p53 wild-type), but basal nuclear localisation of the non-canonical pathway subunit RelB was detectable (Fig. S3). RelB nuclear localisation was dependent upon TBK1 kinase activity as shown by the loss of nuclear RelB stain (Fig. 3a, b) or loss of residency in the nuclear fraction (Fig. 3c) upon treatment with TBK1 inhibitor. Tax1bp1 has previously been demonstrated to inhibit canonical NF-κB signalling by nucleation of an ubiquitin editing complex for canonical NF-κB regulators TRAF6 and RIP1 [24]. However, conflicting reports have demonstrated an NF-κB potentiating effect, albeit downstream of the dysregulation of Tax1bp1 function by the viral oncoprotein Tax [25]. We found that RNAi of *ATG5*, *ULK1*, *TBK1, TAX1BP1* or *NDP52* inhibited RelB nuclear localisation (Fig. 3d-f), implying that a downstream consequence of Tax1bp1 and Ndp52 sequestration by autophagy is engagement of non-canonical NF-κB. Treatment with the autophagy inhibitory compound 3-MA (3-methyl adenine) also ablated RelB nuclear localisation, further supporting these findings (Fig. 3g). It is not clear whether autophagic degradation of cargo in lysosomes, or mere autophagic sequestration of cargo, is required for RelB signalling. While E64d/Pepstatin A treatment had no inhibitory effect on RelB nuclear localisation, a small but significant effect was seen with high doses of chloroquine and a marked inhibitory effect was seen with Bafilomycin A1 (Fig. S4). To confirm the observation that autophagy promotes RelB activity, we assessed changes in gene transcription. Analyses of potential RelB target genes demonstrated that *BIRC3* mRNA levels were robustly downregulated by RelB inhibition (Fig. 3 h, i). Importantly, RNAi of *ATG5*, *ULK1*, *TAX1BP1* and *TBK1* similarly downregulated *BIRC3* mRNA (Fig. 3j).

### RelB Activity Contributes to TBK1 and Autophagy ‘Addiction’

We confirmed previous observations [1], [11] that chronic silencing of *ATG5* or *TBK1*, by lentiviral shRNA targeting, led to a decrease in cell number in A549 cells, as did RNAi of *KRAS* (Fig. S5a-f). Importantly, lentiviral RNAi of *RELB* also led to marked reduction in cell number (Fig. 3k). This suggests TBK1- and autophagy-mediated regulation of RelB may, at least partly, underlie TBK1- and autophagy-addiction.

### Autophagy and Ndp52/Tax1bp1-mediated RelB Signalling can be Independent of Optineurin and Tax1bp1 does not Promote Canonical NF-κB Signalling

**Figure 4:**
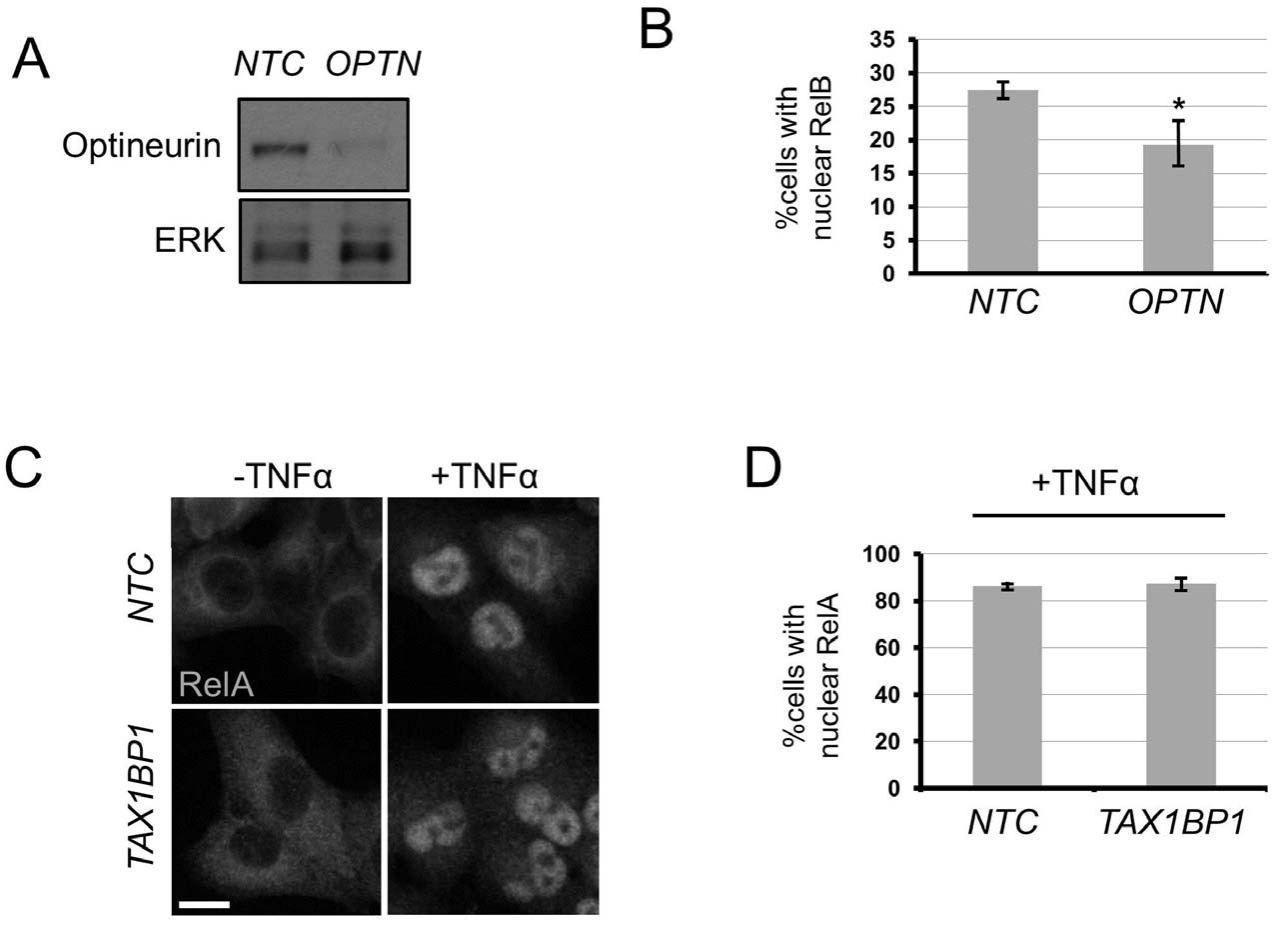
Analysis of roles of Optineurin in RelB signalling and Tax1bp1 in RelA signalling in A549 NSCLC cells. a,b) A549 cells were transfected with indicated siRNA (*NTC* = non-targeting control) for 72 h and a) cell extracts blotted for indicated proteins or b) cells stained and quantified for nuclear RelB (n = 3, ± S.E.M., * = p<0.05). **c,d)** A549 cells were transfected with indicated siRNA (*NTC* = non-targeting control) and stimulated with 5 ng/ml TNF-α for 3 h and c) stained and d) quantified for nuclear RelA (n = 3, ± S.E.M.). Scale bar = 25 µm.

In A549 cells, the other autophagy cargo receptor previously implicated in TBK1 function, Optineurin [16], appears to have limited involvement in RelB signalling. There is only a small, albeit significant, reduction in RelB nuclear localisation upon *OPTN* RNAi (Fig. 4a, b). However, these findings do not exclude a role for Optineurin in different genetic backgrounds or under stress conditions, and these possibilities shall be addressed in future work. In A549 cells, the role of Tax1bp1 in promoting NF-κB signalling is also specific to the novel autophagy-RelB pathway described herein. Stimulation of canonical NF-κB signalling by TNFα treatment, as assessed by nuclear localisation of RelA, is not perturbed by *TAX1BP1* RNAi, at least insofar as no reduction of TNFα-induced nuclear translocation is observed (Fig. 4c, d). Thus the stimulatory role of Tax1bp1 on NF- κB appears to be specific for non-canonical RelB signalling. However, it is important to note that this assay does not address the already well-described inhibitory role of Tax1bp1 on the duration and magnitude of canonical NF- κB signalling [24].

### RelB Activation by Autophagy and Ndp52/Tax1bp1 is also seen in p53-mutated, K-Ras-dependent NSCLC Cells

**Figure 5:**
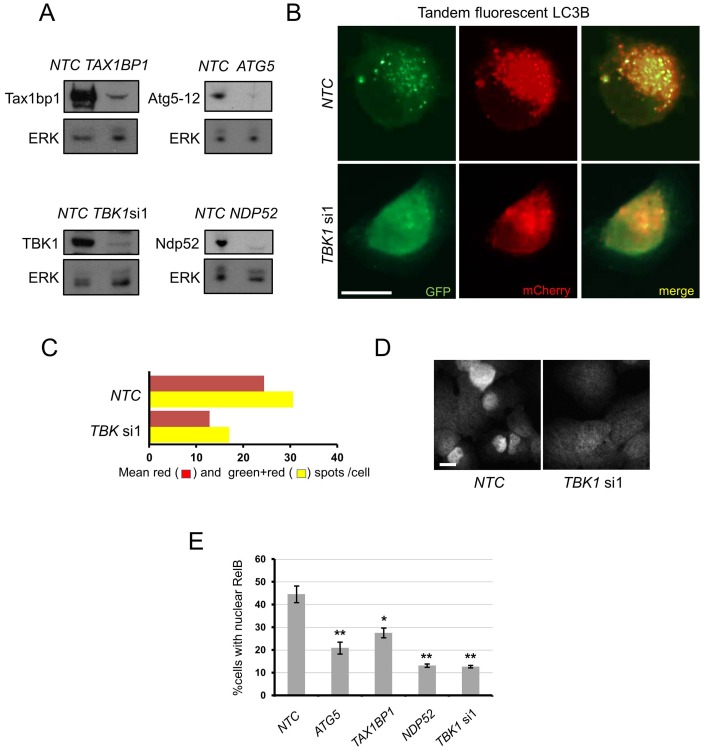
Basal autophagy drives nuclear RelB localisation in NCI-H23 NSCLC cells. a) NCI-H23 cells were transfected with indicated siRNA (NTC = non-targeting control) for 72 h and a) cell extracts blotted for indicated proteins. **b,c)** NCI-H23 tandem-fluorescent LC3B cells were transfected with indicated siRNA and analysed as described in Materials and Methods. **d,e)** NCI-H23 cells were transfected with indicated siRNA for 72 h and cells were d) stained and e) quantified for nuclear RelB (n = 3, ± S.E.M., * = p<0.05, ** = p<0.01). Scale bar = 50 µm in all panels.

It has been proposed that in p53-mutated NSCLC lines, such as NCI-H23, basal canonical NF-κB signalling is much higher than in p53 wild-type lines, such as A549. However, at least in NCI-H23, we still find constitutive engagement of RelB signalling, dependent upon basal autophagy function, suggesting that basal non-canonical and canonical NF-κB signalling may be co-incident (Fig. 5a-e). Specifically, we found that NCI-H23 cells had constitutive autophagy, as assessed with tandem-fluorescent LC3 marker protein, which was downregulated by *TBK1* RNAi (Fig. 5a-c), and that constitutive nuclear localisation of RelB in this cell line was sensitive to RNAi-mediated inhibition of *TBK1*, *ATG5*, *NDP52* or *TAX1BP1* (Fig. 5d, e).

### A Model for Engagement of Basal RelB Signalling in K-Ras-dependent Lung Cancer Cells

**Figure 6:**
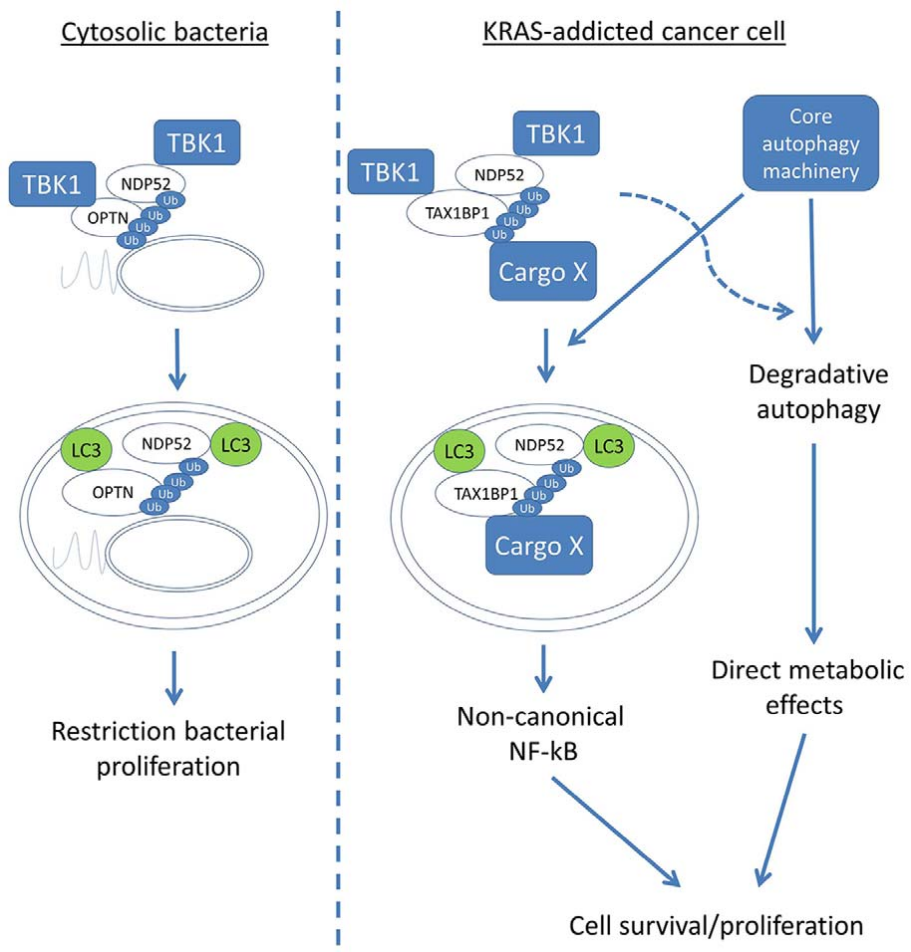
Model of autophagy function in K-Ras addiction versus xenophagy. See text for detailed discussion. Ub = ubiquitin.

The data presented in this manuscript lead to the formulation of the following model (Fig. 6). While the activity of TBK1-binding cargo receptors Optineurin, Ndp52 and, potentially, Tax1bp1, drives autophagy of bacterial cells during xenophagy, a similar TBK1-mediated autophagic sequestration of Tax1bp1 and Ndp52 is driven by basal autophagy in K-Ras-dependent NSCLC cells. This leads to engagement of non-canonical NF-κB signalling and cell proliferation and/or survival. Whether TBK1 also regulates the direct, lysosomal function of autophagy in cellular metabolism is unknown; it also remains to be addressed whether this role of autophagy is experimentally dissociable from selective sequestration of Tax1bp1 and Ndp52. Mechanistically, it is also not clear how TBK1 drives autophagy. However, as this manuscript was in the final stages of revision, the group of Deretic published that phosphorylation of the general function autophagy receptor and facilitator of autophagosome biogenesis, p62/SQSTM1, in the ubiquitin-binding (UBA) domain, may be involved in a novel role for TBK1 in starvation-induced autophagy [26], [27]. Indeed, initial results from this laboratory suggest that A549 cells have basally phosphorylated p62 that is decreased in abundance by TBKi treatment (Fig. S6).

## Discussion

Autophagy is a multifaceted process, enacting changes in cell fate by both non-selective and selective mechanisms [12]. It is likely that the engagement of autophagy in cancer cells acts directly on both metabolism [10], [11], [13] and cell signalling, in an integrated response. It is tempting to speculate that xenophagy is coupled to an innate immune signalling response via non-canonical NF-κB, and this mechanism is simply aberrantly engaged by oncogene-driven autophagy in NSCLC cells. The role of TBK1 in driving autophagy may also explain how autophagy is engaged, despite predicted high basal mTOR activity, in NSCLC cells. Mechanistically, p62 phosphorylation may play a role in this process [26]. However, we speculate that TBK1 is a promiscuous kinase with numerous substrates, and predict that p62, and indeed Optineurin (adjacent to LIR motif) phosphorylation [16], represent merely a subset of functionally relevant targets of TBK1 in autophagy. It is thus possible that different substrates of TBK1 act at different stages of autophagy, either at early steps, as our data would suggest, or at later maturation steps, as has been recently proposed [26].

Generally, NF-κB signalling has been thought to lie upstream of autophagy [28], [29], [30]. However, direct regulation of canonical NF-κB signalling by autophagy has recently been proposed. Hypothesised mechanisms have included sequestration of p62, IκBα, NEMO or Bcl10 [31], [32], [33], [34], [35]. The diversity of mechanisms of autophagy action illustrates the diverse functions of this process; here we add another mechanism of NF-κB regulation, this time in regulation of the non-canonical RelB pathway. Recently, aberrant RelB activity has been identified in lung tumours, fitting with our model [36]. However, p53 wild-type NSCLC might also be dependent, to some degree, upon canonical NF-κB signalling [37], [38]. Indeed, A549 cells (p53 wild-type) are dependent upon c-Rel activity for proliferation and survival [1]. This is not mutually exclusive with our results; an undetectable quantity of nuclear c-Rel could be necessary for proliferation/survival, along with RelB. Indeed, the context of p53-mutated NSCLC, where engagement of canonical NF-κB signalling is much more evident [39], will be a good arena to dissect such interplay. Interestingly, we have shown in this manuscript that in at least some cell lines with p53 mutation, and reported prominent basal canonical NF-κB signalling (e.g. NCI-H23), that basal, autophagy-driven RelB nuclear localisation can be nonetheless detected. Future investigation of the autophagy cargoes brought to autophagosomal membranes by Tax1bp1 and Ndp52, ubiquitinated or otherwise, will also shed light upon the mechanism by which these cargo receptors are involved in the mediation of non-canonical NF-κB signalling. Proteomic experiments to determine the identity of these cargoes are underway in our laboratory.

Given the interest in developing TBK1 inhibitors for cancer treatment, we need to understand in detail the consequences of loss of the pathway described here. In the *in vivo* setting, the effects of RelB regulation by autophagy and TBK1 are unlikely to be wholly cell autonomous, given the well-established role of NF-κB signalling in controlling cell-cell communication and inflammation. Interestingly, *TAX1BP1* may regulate intestinal tumourigenesis in mouse models of cancer [40]. Also, germline *TAX1BP1* knockout mice display an organ-specific inflammatory phenotype [41]. It is possible that these observations may relate to the role for Tax1bp1 described here or, alternatively, to the better described role in repressing canonical NF-κB signalling [24]. Finally, targeting of autophagy is also proposed as a means of cancer treatment, at least in some genetic backgrounds. The data here suggest caution in extrapolating the results of genetic inhibition of autophagy by targeting autophagosome formation and sequestration, to the action of drugs that target lysosomal function, such as chloroquine, protease inhibitors or bafilomycin A1. It is not clear that agents such as these will effect all the changes, such as NF-κB activity, that targeting the early stages of autophagy will, and *vice-versa*.

## Materials and Methods

### Cell Lines and Plasmids

All cells were cultured in DMEM (Sigma) +10% FCS (Sigma) under 5% CO2 at 37°C. HEK293FT cells were from Invitrogen. A549 cells used were obtained from Institute of Cancer Research (London, UK) and identity verified by microsatellite genotyping (Health Protection Agency, UK). NCI-H23 cells were purchased directly from ATCC. A549 and NCI-H23 cells were derivatised to express Ecotropic Receptor using pEcoR-neo and selection in G418. Retrovirus to make stable infectants was generated in Phoenix-Eco cells by standard techniques. These viruses were: **pBabe GFP-LC3B puro** [42]: rat LC3B cDNA fused N-terminally to GFP. **pBabe GFP-LC3BΔG->A puro**: above plasmid encoding protein truncated so C-ter Gly exposed by processing of C-terminus by Atg4 is constitutively exposed. This C-ter Gly is mutated to Ala. **pBabe tfLC3B puro**: pBabe puro with tandem fluorescent LC3B [21] cloned in NheI (blunt)/SalI into SnaBI/SalI site. **pWZL GFP-TBK1 HYGRO**: pWZL GFP DEST IRES HYGRO vector (unpublished) with full length TBK1 fused to C-terminus of GFP by recombination with pDONR 223 TBK1 (unpublished) by Gateway technology (Invitrogen). **pBabe mCherry LC3C BLAST**: pBabe Cherry DEST BLAST vector (unpublished) with full length LC3C fused to C-terminus of mCherry by recombination with pDONR 223 LC3C [43] by Gateway technology (Invitrogen). **MSCV FLAG-HA-LC3B:** As described in literature {Behrends, 2010 #4}. **MSCV FLAG-HA- TAX1BP1 (WT and LIR mutants)**: TAX1BP1 cDNA was cloned into pDONR 223 by standard Gateway cloning techniques using pcDNA 3.1 myc-TAX1BP1 (below) as a template. TAX1BP1 cDNA was then Gateway cloned into MSCV NTAP IRES PURO [43]. Site-directed mutagenesis was performed in pDONR prior to recombination into MSCV NTAP IRES PURO to produce cDNA encoding TAX1BP1 with mutations in the putative canonical or non-canonical LIR motif (mutations as described in text). All mutagenized cDNAs were fully sequenced.

Other plasmids used in this study were: **pcDNA 3.1 myc-His:** Invitrogen, **pcDNA 3.1 myc-TAX1BP1:** myc-TAX1BP1 full-length cDNA (NCBI isoform 1) was PCR amplified with the following primers from an IMAGE clone and cloned BamHI-XhoI into pCDNA 3.1 myc-His: GGA TCC GCC ACC ATG GAG CAG AAG CTG ATT TCC GAG GAG GAC CTG ACA TCC TTT CAA GAA GTC CCA TTG CAG ACT TC and CTC GAG CTA GTC AAA ATT TAG AAC ATT CTG ATC AAA ATG GGT C. The resultant cDNA encodes the 307I variant of this protein (common polymorphism). **pDEST15-empty vector** (for expression of GST control protein): created by inserting an in-frame stop codon containing cassette into pDEST15 by Gateway cloning from a specially constructed pDONR223 plasmid. **pDEST15-LC3B, pDEST15-LC3C** and **pDEST15-GABARAP** (for expression of GST fusion proteins) were created by recombination with pDONR 223 LC3B, pDONR 223 LC3C or pDONR 223 GABARAP [43] by Gateway technology (Invitrogen).

### Chemicals

Chloroquine was obtained from Sigma and maintained as a frozen stock solution in PBS. 3-MA was obtained from Sigma and fresh solutions made in DMEM prior at working concentration prior to application to cells. TNFα was obtained from Sigma and stored in frozen aliquots in aqueous solution. TBKi (MRT68601, Fig. S1) was obtained from MRC Technology and maintained as a frozen stock solution in DMSO. Bafilomycin A1 was obtained from Sigma and stored as a frozen stock solution in DMSO. Pepstatin A was obtained from Sigma and stored as a stock solution in ethanol. E64d was obtained from Calbiochem and stored as a frozen stock solution in DMSO.

### Antibodies

The antibodies used in this study are as follows: TBK1 (Rabbit AOW9 monoclonal, Millipore), Phospho-Ser172–TBK1 (BD Pharmingen), ATG5 (for ATG5-12, Rabbit polyclonal, Cell Signalling Technology), ULK1 (Rabbit polyclonal A705, Cell Signalling Technology), LC3B (Immunoblot, Rabbit monoclonal D11, Cell Signalling Technology), LC3B (Immunofluorescence, Mouse monoclonal 2G6, Nanotools), GABARAP (Rabbit polyclonal Ap1821a, Abgent), ERK (Rabbit polyclonal 9102, Cell Signalling Technology), RelB (Rabbit monoclonal C1E4, Cell Signalling Technology), Histone H3 (H3, Mouse monoclonal 6-6-2, Millipore), Tax1bp1 (Rabbit poly HPA024432, Sigma HPA), Ndp52 (Rabbit poly ab68588, Abcam), p62 (mouse 3/p62 monoclonal, BD Pharmingen), myc tag (Rat JAC6 monoclonal, AbD Serotec [TBK1 coIP experiments] or Mouse 4A6 monoclonal, Millipore [GST pulldown experiments]), c-Rel (Rabbit polyclonal, 4727,Cell Signalling Technology), RelA (Rabbit monoclonal C22B4, Cell Signalling Technology), α-tubulin (Mouse monoclonal DM1A, Sigma), HA (Rat monoclonal 3F10, Roche), GST (GST-2 mouse monoclonal, Sigma), Optineurin (Rabbit polyclonal Item no. 100000, Cayman Chemical), K-Ras (Mouse monoclonal 3B10-2F2, Sigma). Anti-phospho-S403-p62 was a kind gift of Nobuyuki Nukina (RIKEN, Japan). Phospho-specific antibodies to IRF3, RelA and JNK were obtained from Cell Signalling Technology.

### siRNA Transfection

In 35 mm dishes, 0.8×10^5^ A549 or 1.2×10^5^ NCI-H23 were plated overnight. Cells were transfected for 8 h with Oligofectamine (Invitrogen) and 10 nM siRNA (A549) or 20 nM siRNA (NCI-H23), according to the manufacturer’s instructions. siRNA duplexes were as follows: *NTC* (siGenome non-targeting control 1, Dharmacon, sequence not provided by manufacturer), *TBK1* si1 (Ambion, GGTTTGGCTCTTTAACCAT), *TBK1* si2 (Hs_TBK1_6 Validated siRNA, Qiagen, sequence not provided by manufacturer) *ATG5* (siGenome duplex 004374-03, Dharmacon, CATCTGAGCTACCCGGATA), *ULK1* (Hs_ULK1_5 Validated siRNA, Qiagen, sequence not provided by manufacturer), *TAX1BP1* (Hs_TAX1BP1_8 Validated siRNA, Qiagen, CAGTCTTTGGCTTATCAAT), *NDP52* (Hs_CALCOCO2_3 siRNA, Qiagen, TCAGGGACTTAGGTAATTT) and *OPTN* (Hs_OPTN_2 siRNA, Qiagen, CAGCGGAATATTCCGATTCAT).

### Tandem Fluorescent LC3B Assay

Cells stably expressing tandem fluorescent LC3B, from pBabe puro tfLC3B, were treated as indicated in text and fixed and mounted on glass slides. Multiple fields were imaged using wide-field fluorescence microscopy, capturing images of both mCherry fluorescence and GFP fluorescence with separated, optimised exposures in separate channels. Visual inspection determined that GFP positive puncta, virtually without exception, were also scored as mCherry positive in corresponding images. The number of mCherry positive puncta was counted across all cells and then the number of GFP positive puncta was likewise summated. The total number of ‘green+red’ autophagic structures were given by the number of mCherry positive puncta, while ‘red’ late autophagic structure numbers were derived by subtracting by GFP-positive mCherry-negative puncta numbers. At least 100 cells were counted for each condition.

### Lentiviral shRNA Infection and Assays

Virus was produced in 293FT cells using pCMV-VSVG and pCMVRΔ8.2 plasmids according to established protocols [1]. Equivalent titres were used in control and experimental groups, in the range of 5< M.O.I. <10. 24 h post-infection, cells were selected for 48 h in 2.5 µg/ml puromycin. For analysis beyond 72 h time point, cells were always replated into larger vessels, in standard medium (no puromycin) to prevent attainment of confluency in controls. Cell counting was performed by trypsinisation of live adherent cells and use of a haemocytometer. Numbers were normalised so that the mean of independent experiments would equal 1 for control treatments. Hairpins used were in pLKO.1 puro, targeting sequences as follows: *NTC* (TAA GGC TAT GAA GAG ATA C), *GFP* (ACA ACA GCC ACA ACG TCT ATA), *RELB* (GGT GCA GAA AGA GGA CAT ATC), *ATG5-1* (ACT CAC ATA CAG TAG ATC ACT) *ATG5-2* (GCT AGC TGG CTG TCC ATA TTG) *TBK1-1* (GCA GAA CGT AGA TTA GCT TAT) *TBK1-2* (GCG GCA GAG TTA GGT GAA ATT), *KRAS-1* (CCT CGT TTC TAC ACA GAG AAA), *KRAS-2* (GAG GGC TTT CTT TGT GTA TTT).

### Protein Extraction and Immunoblotting

All cell extracts were made in RIPA buffer (50 mM Tris-HCl, pH 7.5, 0.5% Na deoxycholate, 1% Triton-X-100, 150 mM NaCl, complete protease inhibitors +1 mM EDTA (Roche), 2 mM Na orthovanadate 25 mM Na beta-glycerophosphate, 10 mM NaF, 10 mM Na pyrophosphate), with the exception of lysates for LC3B or GABARAP immunoblotting, which were extracted directly in 2X SDS-PAGE sample buffer (4% SDS, 20% glycerol, 0.125 M Tris-HCl pH 7.5, 10% w/v beta-mercaptoethanol, bromophenol blue). Gel electrophoresis was performed with MOPS/Tris-Bis buffer system on gradient gels (Invitrogen). Immunodetection techniques were performed according to standard procedures using enhanced chemiluminescence detection. Nitrocellulose membrane for blotting was used in all experiments except for LC3B and GABARAP immunodetection, where PVDF was used.

### Immunostaining and Microscopy

For immunofluorescence, cells were grown on glass coverslips and fixed in 4% paraformaldehyde. Fluorescent protein fusions were imaged on cells mounted at this point. Alternatively, if further immunostaining was to be performed, cells were generally permeabilised with 0.25% Triton-X-100. In cases where endogenous LC3B immunodetection was performed, permeabilisation was for ten minutes at −20°C with methanol. Cells were visualised on an Olympus BX51 widefield fluorescence microscope with constant acquisition time and illumination intensity across samples sets, under 40 X magnification, using a DP71 digital capture device, or using an Olympus FV1000 confocal microscope. Quantification of puncta or nuclear RelB was always performed by blind scoring. For merge images for colocalisation, adjustment of relative level and curves for red or green channel was applied across the whole image after acquisition using Paint.NET software. For tandem fluorescent LC3B scoring, no adjustments were made.

### GST Pull-down Assays

pDEST15-empty (GST only control), or pDEST15 expressing GST fusions of ubiquitin-like proteins, were induced for 3 hours with 0.1 mM IPTG in Rosetta 2 DE3 bacteria. After resuspension of bacterial pellet in IP buffer (50 mM Tris-HCl, pH 7.5, 0.5% IGEPAL CA-630, 150 mM NaCl, complete protease inhibitors +1 mM EDTA [Roche]), cells were broken open by sonication, and a soluble GST-positive fraction obtained by pelleting bacterial debris. This fraction was used to pull-down mammalian control lysates or lysates containing overexpressed myc-Tax1bp1. These lysates were obtained from 293FT cells transfected for 48 h with pcDNA 3.1 myc-His or pcDNA3.1 myc-Tax1bp1, respectively, using Lipofectamine 2000 (Invitrogen) and 1 µg of DNA (in 35 mm dish), according to manufacturer’s instructions. Lysates were made with IGEPAL IP buffer (below). Pull-down was performed by coincubation overnight at 4°C and then purification of complexes using Glutathione-Sepharose 4B and use of IGEPAL IP buffer for washes.

### Nuclear Fraction Enrichment

‘Quick’ fractionation was performed to minimise loss of nuclear NF-κB. Briefly, cells were lysed in ice-cold cytosolic extraction buffer (10 mM Hepes pH 7.9, 1.5 mM MgCl_2_, 10 mM KCl, 0.05% IGEPAL CA-630, 0.5 mM DTT, complete protease inhibitors +1 mM EDTA (Roche), 2 mM Na orthovanadate, 25 mM Na beta-glycerophosphate, 10 mM NaF). A 3000 rpm pellet was obtained from a spin in a benchtop microcentrifuge at 4°C. This pellet (nucleus and some membrane fragments) was washed briefly with ice-cold extraction buffer and then immediately lysed in 2X SDS-PAGE sample buffer.

### Immunoprecipitation

Briefly, cells were lysed in IGEPAL IP buffer (50 mM Tris-HCl, pH 7.5, 0.5% IGEPAL CA-630, 150 mM NaCl, complete protease inhibitors +1 mM EDTA (Roche), 2 mM Na orthovanadate 25 mM Na beta-glycerophosphate, 10 mM NaF, 10 mM Na pyrophosphate), either directly or 48 h post-transfection with indicated plasmids by Lipofectamine 2000 (Invitrogen) and 1 µg of DNA (in 35 mm dish) according to manufacturer’s instructions. Lysates were subject to immunoprecipitation for 4 hours using 1 µg of indicated antibody, for endogenous protein bait, pre-bound to protein A/G agarose (Roche), or anti-myc magnetic beads, for transfected protein bait (Cell Signalling Technology), and three washes in lysis buffer. Protein was eluted from beads by boiling in 2X SDS-PAGE sample buffer.

### qRT-PCR

Two-step quantitative real-time PCR was performed on RNA purified using Qiagen RNeasy kit and reverse transcribed using first strand cDNA synthesis kit (Applied Biosystems). SYBR green hot-start PCR kit (Finnzymes) was used for second reaction step on a Corbett Rotor-Gene 3000 cycler. Primer sequences used for qRT-PCR were as follows. *18S*: GTAACCCGTTGAACCCCATT and CCATCCAATCGGTAGTAGCG *BIRC3*: GCCCTCTAGTGTTCTAGTTAATCC and TACTCACACCTTGGAAACCAC. All *BIRC3* readings were normalised to *18S*.

### Statistical Methods

All calculations of significance were done using one-tailed Student’s t-test, or ANOVA with post-hoc analysis of significance for greater than two experimental conditions, all using Microsoft Excel with WinSTAT plugin.

## Supporting Information

Figure S1 **Characterisation of the TBK inhibitor MRT68601. a)** Chemical structure of MRT68601, the TBK kinase inhibitory compound used in this study. **b)** Kinase selectivity profile for the TBKi, MRT68601. Kinase selectivity profiling was performed at the Protein Phosphorylation Unit, University of Dundee (James Hastie and Hilary McLauchlan) for key kinases. TBKi is a potent inhibitor of both TBK1 and to a lesser extent IKKε. With the exception of MARK3, the compounds show at least 10-fold selectivity over TBK1 against all of the other kinases tested. **c,d)** Inhibitory effects of TBKi (MRT68601) on LPS-stimulated IRF3 phosphorylation and IFN-β release in RAW264.7 macrophages. c) Cells were cultured in 6-well plates (1×10^6^/well) and pre-incubated with 10-fold dilutions of TBKi for 30 minutes, prior to stimulation with LPS (1 mg/ml) for 1 hour and collection of lysates. Lysates were immunoblotted with indicated phospho-specific antibodies to IRF3, RelA, JNK or TBK1. d) Cells were cultured in 6-well plates (1×10^6^/well) and pre-incubated with 10-fold dilutions of TBKi for 30 minutes, prior to stimulation with LPS (1 mg/ml) for 2 hours and subsequent nuclear fractionation and ELISA determination of DNA-bound IRF3 (TransAM™, Active Motif). Data presented as mean ± S.D., n = 2 independent repeats.
(TIF)

Figure S2 **Validation of Tax1bp1 and Ndp52 protein complex formation with TBK1 and the location of proposed LIR motifs in Tax1bp1. a)** 293FT cells were transfected with either pcDNA 3.1 myc-His or pcDNA 3.1 myc-Tax1bp1 plasmid. 48 h later cells were lysed in IGEPAL IP buffer and supernatants (input) immunoprecipitated with anti-myc antibody and both input and immunoprecipitate samples blotted with indicated antibodies. **b)** Exponentially growing A549 cells in the basal state were lysed in IGEPAL IP buffer and subjected to immunoprecipitation with either non-specific rabbit IgG or anti-Ndp52 and both input and immunoprecipitate samples blotted with indicated antibodies. Arrow indicates Ndp52, asterisk indicates IgG heavy chain. **c)** Alignment of human Tax1bp1 and Ndp52 sequences showing potential canonical and non-canonical LIR motifs in the N-terminal regions of Tax1bp1.
(TIF)

Figure S3 **Basal localisation of c-Rel, RelA and RelB in A549 cells.** A549 cells growing exponentially, in the basal state, were fixed and stained with indicated antibodies. Scale bar = 50 µm.
(TIF)

Figure S4 **Effect of lysosomal inhibitors on RelB nuclear localisation. a-c)** A549 cells were treated with a) DMSO or 0.1 µM Bafilomycin A1 (BafA1) for 48 h, b) PBS or 5 µM chloroquine (CQ) for 24 h or c) DMSO/Ethanol (EtOH) vehicle control or 10 µg/ml each E64d and Pepstatin A (PepA) for 48 h. All cells were then stained and quantified for nuclear RelB (n = 3, ± S.E.M., * = p<0.05, ** = p<0.01, # = not significant).
(TIF)

Figure S5 **Requirement of autophagy, TBK1 and K-Ras for proliferation and/or survival of A549 cells. a,b)** A549 cells were infected with indicated lentiviral supernatants and a) cell extracts blotted for indicated proteins at 96 hours post infection or b) cell numbers counted at 120 h (n = 3, ± S.E.M.). **c,d)** A549 cells were treated with indicated concentrations of MRT68601 (TBKi) for 72 hours and c) images taken by phase contrast microscopy or d) viable cells counted (n = 3, ± S.E.M.). **e,f)** A549 cells were infected with indicated lentiviral supernatants and e) cell extracts blotted for indicated proteins at 72 hours post infection or f) cell numbers counted at 120 h (n = 3, ± S.E.M.).
(TIF)

Figure S6 **p62 phosphorylation downstream of TBK1 activity.** A549 cells were treated overnight with 10 µM TBKi and stained with anti-phospho-Ser403-p62 antibody. Scale bar = 50 µm.
(TIF)
