# Supplementary figures and images for: TBK1 Kinase Addiction in Lung Cancer Cells Is Mediated via Autophagy of Tax1bp1/Ndp52 and Non-Canonical NF-κB Signalling
Source: PLoS One. 2012 Nov 29;7(11):e50672. doi: 10.1371/journal.pone.0050672 (PMC3510188; doi:10.1371/journal.pone.0050672)

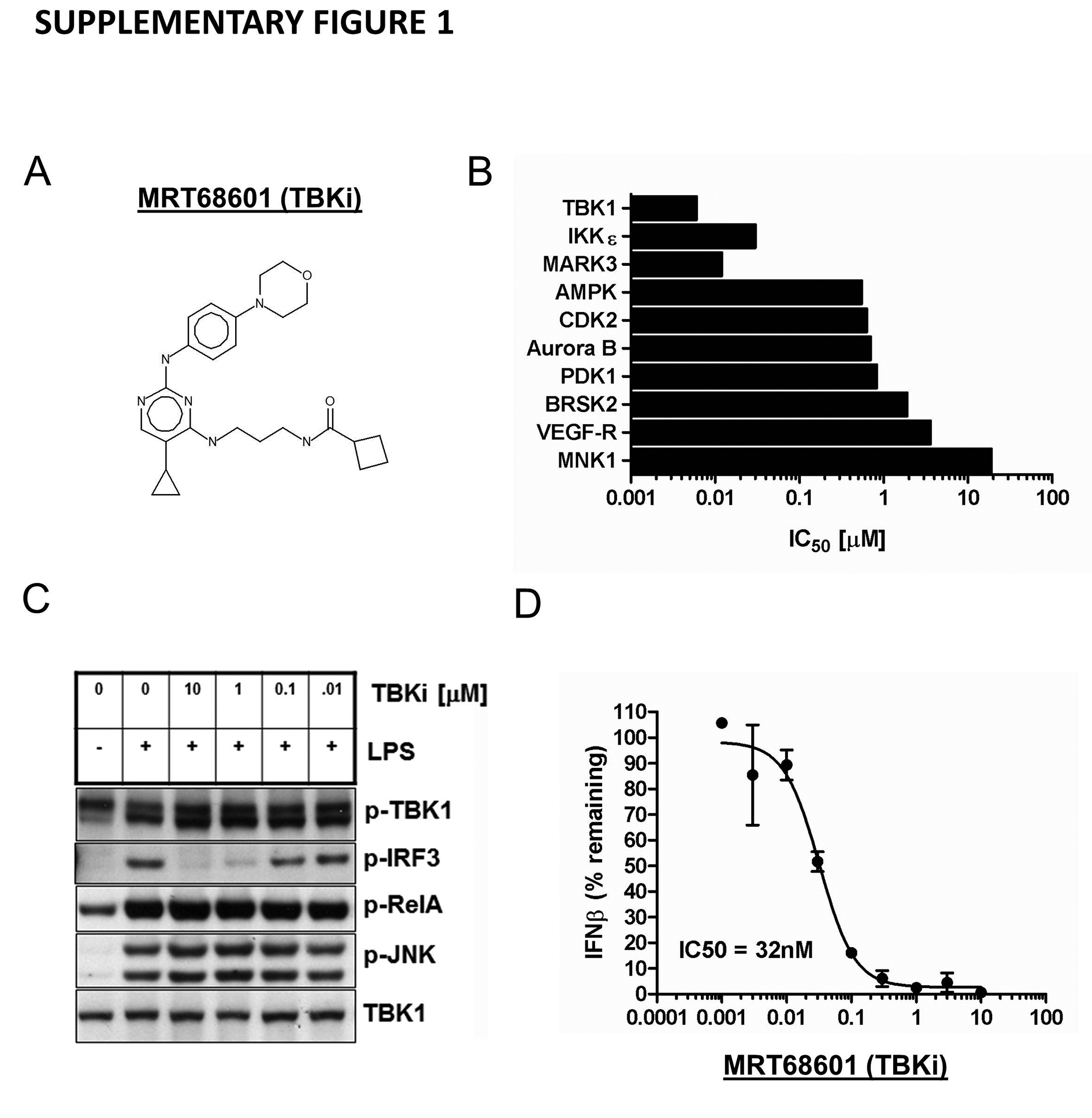

Supplement: Figure S1 — Characterisation of the TBK inhibitor MRT68601. a) Chemical structure of MRT68601, the TBK kinase inhibitory compound used in this study. b) Kinase selectivity profile for the TBKi, MRT68601. Kinase selectivity profiling was performed at the Protein Phosphorylation Unit, University of Dundee (James Hastie and Hilary McLauchlan) for key kinases. TBKi is a potent inhibitor of both TBK1 and to a lesser extent IKKε. With the exception of MARK3, the compounds show at least 10-fold selectivity over TBK1 against all of the other kinases tested. c,d) Inhibitory effects of TBKi (MRT68601) on LPS-stimulated IRF3 phosphorylation and IFN-β release in RAW264.7 macrophages. c) Cells were cultured in 6-well plates (1×10^6^/well) and pre-incubated with 10-fold dilutions of TBKi for 30 minutes, prior to stimulation with LPS (1 mg/ml) for 1 hour and collection of lysates. Lysates were immunoblotted with indicated phospho-specific antibodies to IRF3, RelA, JNK or TBK1. d) Cells were cultured in 6-well plates (1×10^6^/well) and pre-incubated with 10-fold dilutions of TBKi for 30 minutes, prior to stimulation with LPS (1 mg/ml) for 2 hours and subsequent nuclear fractionation and ELISA determination of DNA-bound IRF3 (TransAM™, Active Motif). Data presented as mean ± S.D., n = 2 independent repeats. (TIF) [file pone.0050672.s001.tif]

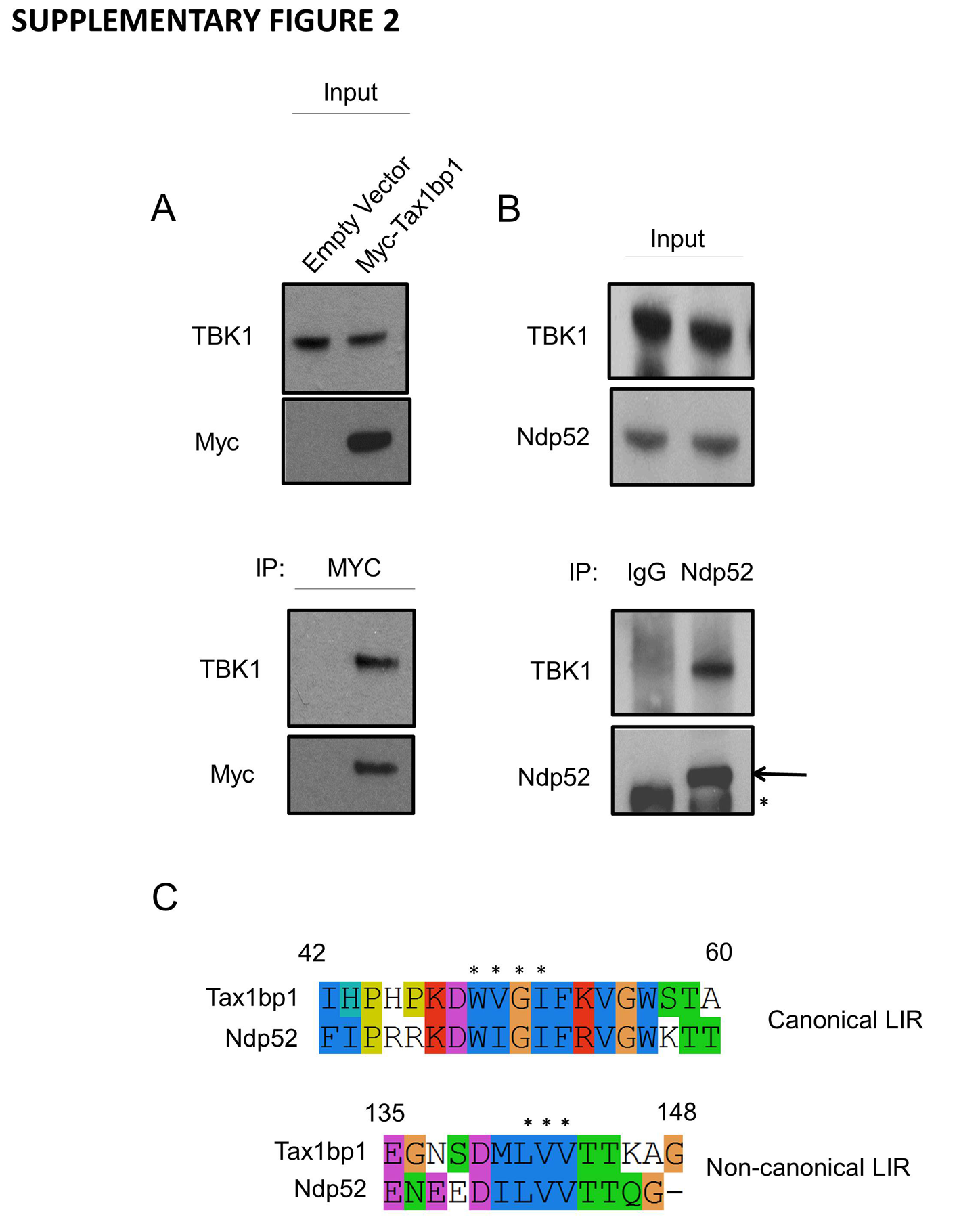

Supplement: Figure S2 — Validation of Tax1bp1 and Ndp52 protein complex formation with TBK1 and the location of proposed LIR motifs in Tax1bp1. a) 293FT cells were transfected with either pcDNA 3.1 myc-His or pcDNA 3.1 myc-Tax1bp1 plasmid. 48 h later cells were lysed in IGEPAL IP buffer and supernatants (input) immunoprecipitated with anti-myc antibody and both input and immunoprecipitate samples blotted with indicated antibodies. b) Exponentially growing A549 cells in the basal state were lysed in IGEPAL IP buffer and subjected to immunoprecipitation with either non-specific rabbit IgG or anti-Ndp52 and both input and immunoprecipitate samples blotted with indicated antibodies. Arrow indicates Ndp52, asterisk indicates IgG heavy chain. c) Alignment of human Tax1bp1 and Ndp52 sequences showing potential canonical and non-canonical LIR motifs in the N-terminal regions of Tax1bp1. (TIF) [file pone.0050672.s002.tif]

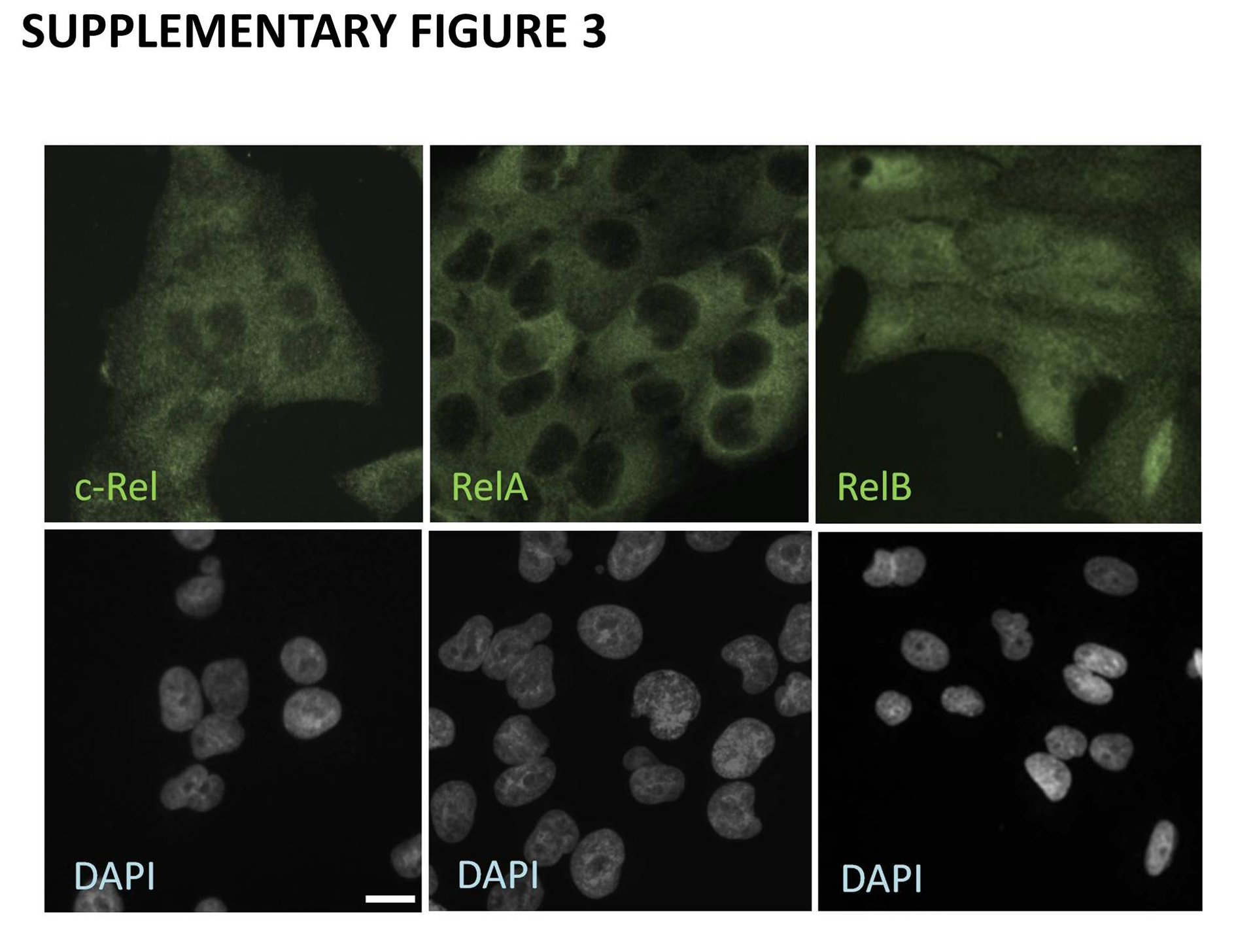

Supplement: Figure S3 — Basal localisation of c-Rel, RelA and RelB in A549 cells. A549 cells growing exponentially, in the basal state, were fixed and stained with indicated antibodies. Scale bar = 50 µm. (TIF) [file pone.0050672.s003.tif]

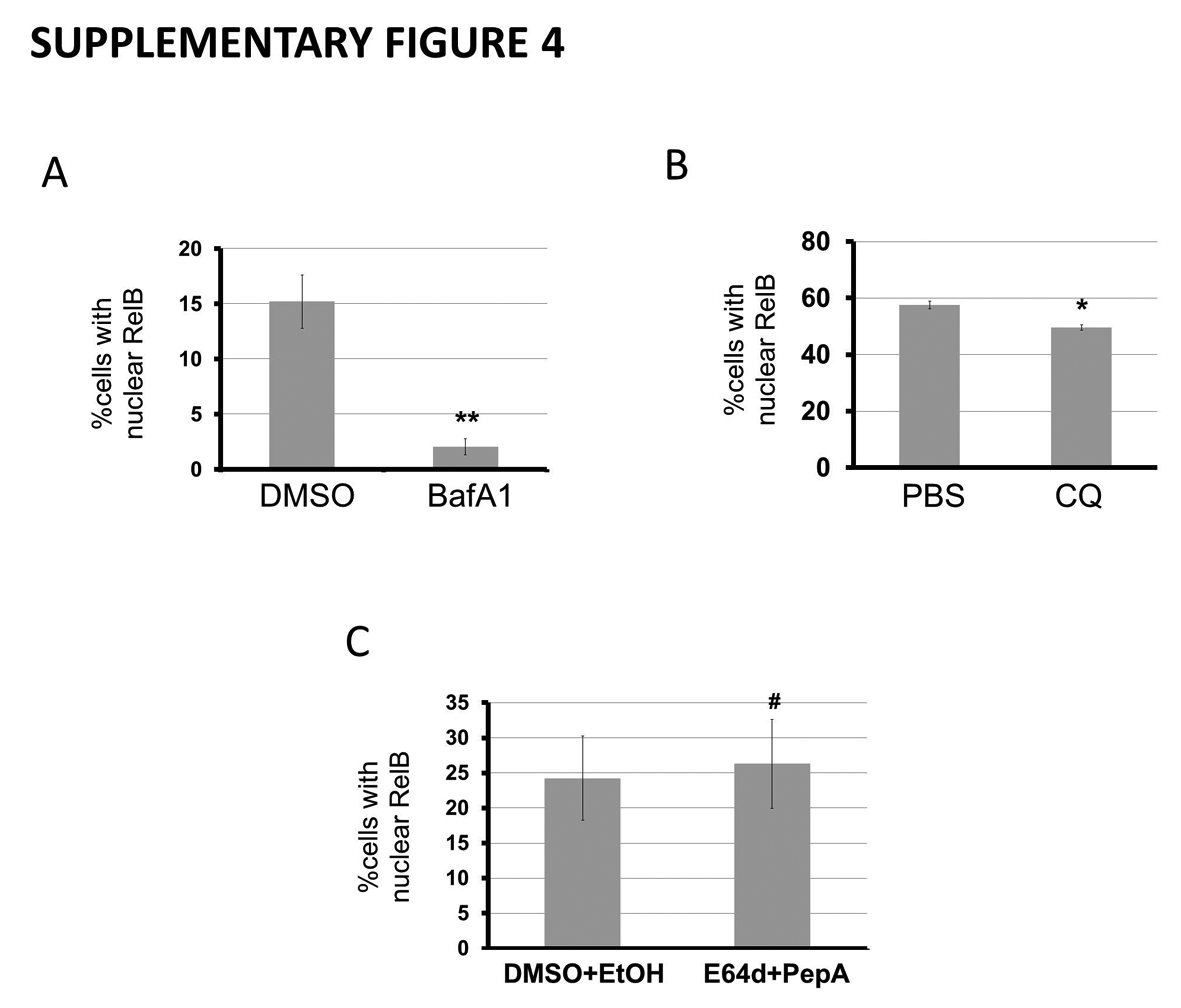

Supplement: Figure S4 — Effect of lysosomal inhibitors on RelB nuclear localisation. a-c) A549 cells were treated with a) DMSO or 0.1 µM Bafilomycin A1 (BafA1) for 48 h, b) PBS or 5 µM chloroquine (CQ) for 24 h or c) DMSO/Ethanol (EtOH) vehicle control or 10 µg/ml each E64d and Pepstatin A (PepA) for 48 h. All cells were then stained and quantified for nuclear RelB (n = 3, ± S.E.M., * = p<0.05, ** = p<0.01, # = not significant). (TIF) [file pone.0050672.s004.tif]

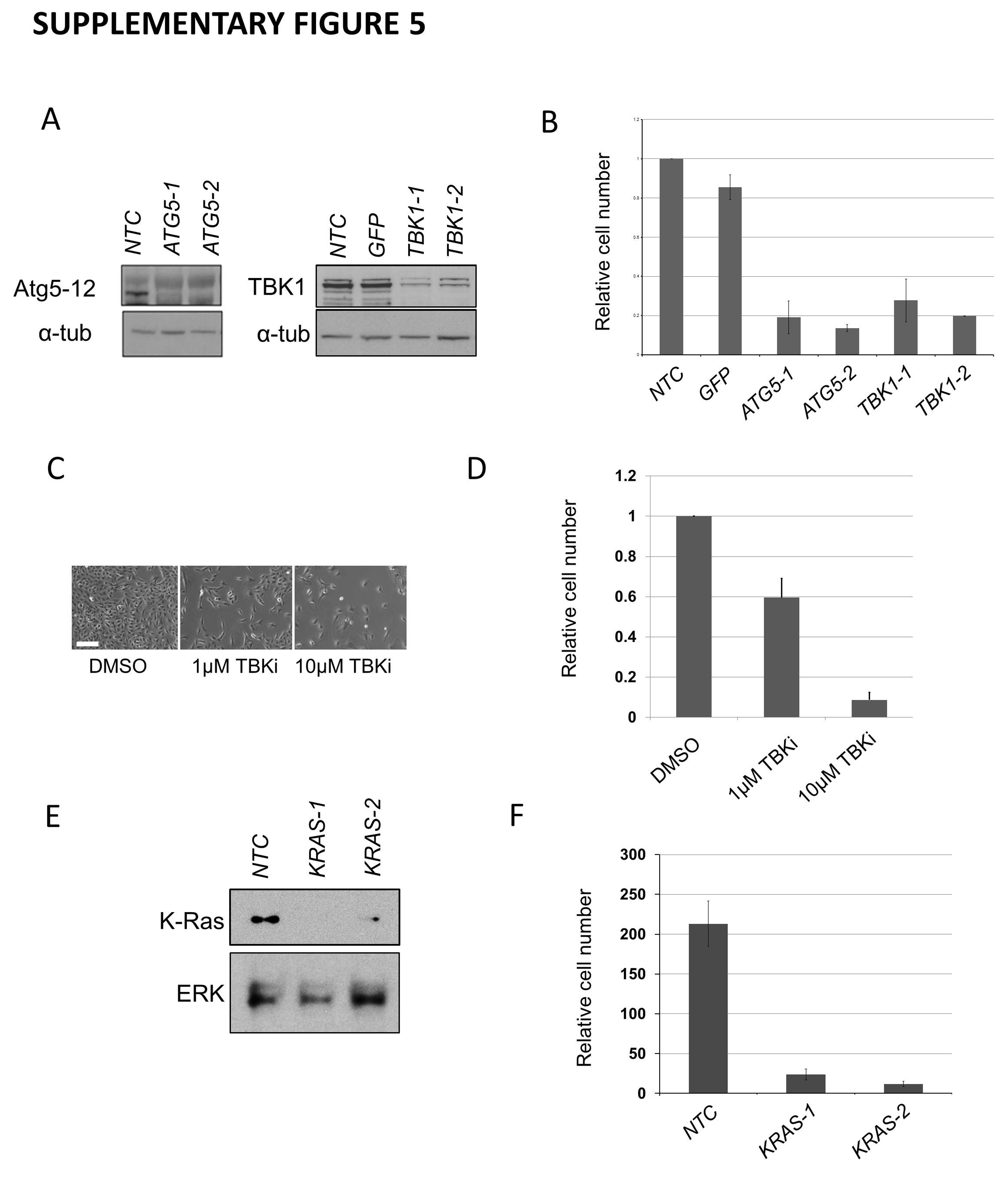

Supplement: Figure S5 — Requirement of autophagy, TBK1 and K-Ras for proliferation and/or survival of A549 cells. a,b) A549 cells were infected with indicated lentiviral supernatants and a) cell extracts blotted for indicated proteins at 96 hours post infection or b) cell numbers counted at 120 h (n = 3, ± S.E.M.). c,d) A549 cells were treated with indicated concentrations of MRT68601 (TBKi) for 72 hours and c) images taken by phase contrast microscopy or d) viable cells counted (n = 3, ± S.E.M.). e,f) A549 cells were infected with indicated lentiviral supernatants and e) cell extracts blotted for indicated proteins at 72 hours post infection or f) cell numbers counted at 120 h (n = 3, ± S.E.M.). (TIF) [file pone.0050672.s005.tif]

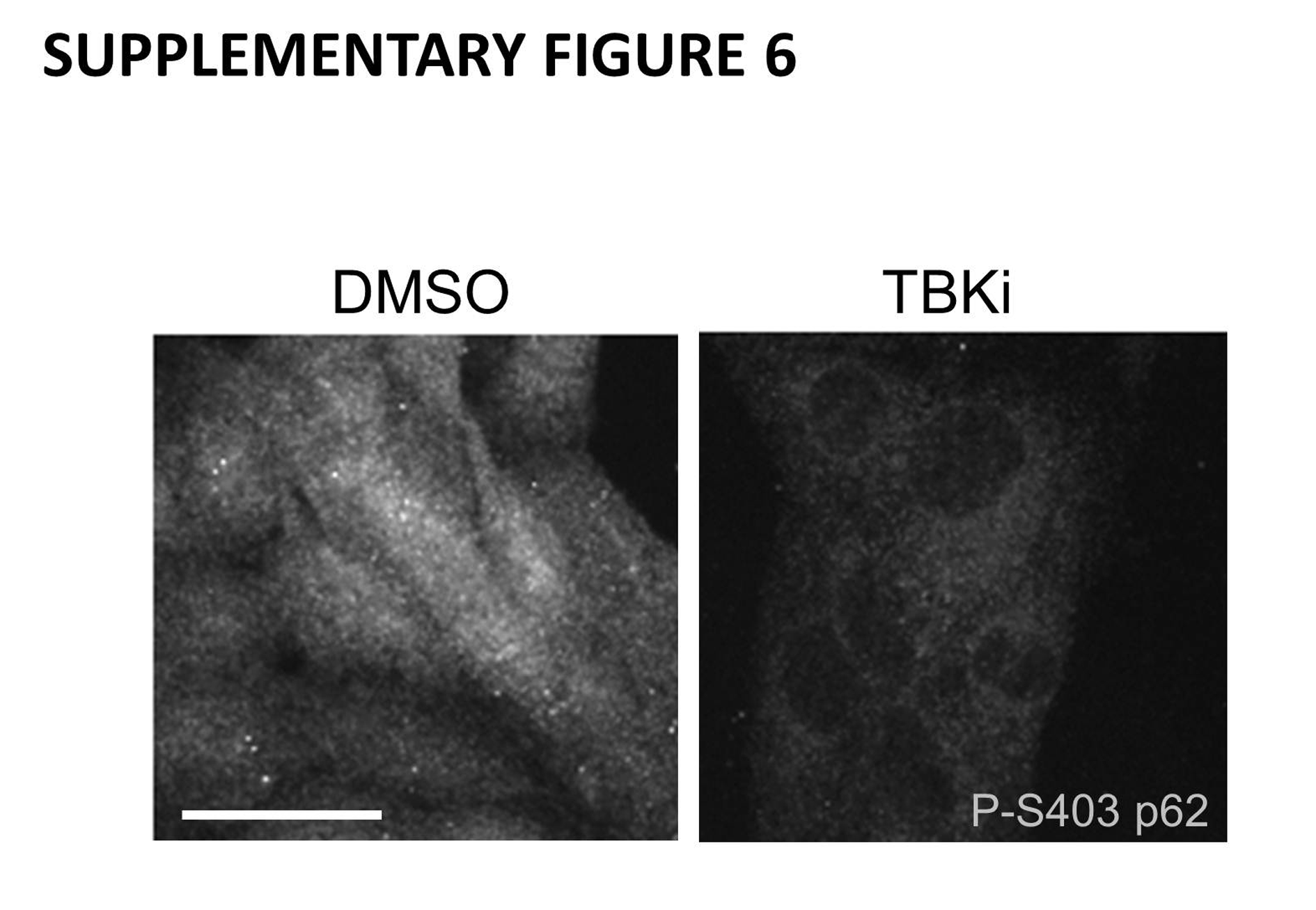

Supplement: Figure S6 — p62 phosphorylation downstream of TBK1 activity. A549 cells were treated overnight with 10 µM TBKi and stained with anti-phospho-Ser403-p62 antibody. Scale bar = 50 µm. (TIF) [file pone.0050672.s006.tif]
